# An age and gender stratified interview on emotional experiences and coping of Chinese migrants in Canada amidst the pandemic

**DOI:** 10.1186/s40359-025-02993-6

**Published:** 2025-07-04

**Authors:** Lixia Yang, Yating Ding, Miao Wang, Jingya Xie, Weiguo Zhang, Peizhong Peter Wang

**Affiliations:** 1 Toronto Metropolitan University, Toronto, ON Canada; 2 Ted Rogers School of Management, Toronto Metropolitan University, Toronto, ON Canada; 3 Department of Sociology, University of Toronto Mississauga, Mississauga, ON Canada; 4 Division of Population Health and Applied Health Sciences, Faculty of Medicine, Memorial University, St. John’s, NL Canada; 5 Dalla Lana School of Public Health, University of Toronto, Toronto, ON Canada; 6 Centre for New Immigrant Well-Being, Toronto, Canada

**Keywords:** Emotional experiences, Coping, Chinese migrants, Psychological well being, COVID-19 pandemic

## Abstract

The COVID-19 pandemic had a profound impact on psychological wellbeing. The current study aims to identify common emotional experiences and coping strategies among Chinese migrants in Canada based on a semi-structured interview with a purposive sample of 20 young (aged 21–34), 21 middle-aged (aged 42–57), and 20 older adults (aged 65–85). Each age group was approximately split in half by gender. The qualitative analysis of the data identified some common positive (e.g., self-oriented, other-oriented and situational) and negative emotional experiences (e.g., sadness, loneliness, fear, and anger) towards the pandemic. Coping strategies were categorized into three themes: behavioural (e.g., exercise, relaxation), social (e.g., social support), and cognitive (e.g., reappraisal). The supplementary quantitative analysis showed that women reported more negative than positive experiences, while older adults (women particularly) endorsed differentially more behavioural coping. This suggests that women are likely to be emotionally vulnerable and aging is likely to be associated with more adaptive coping among this population in the context of the pandemic.

**Supplementary Information:**

The online version contains supplementary material available at 10.1186/s40359-025-02993-6.

## Introduction

The COVID-19 outbreak has left tremendous impacts on individual and collective psychological wellbeing across the world [1]. It has also increased health inequalities, with a particularly severe impact on women, younger adults, those with disabilities, and racialized minority groups [e.g., [2–6]]. While lockdown and social/physical distancing measures effectively prevented the spread of the COVID-19 virus, their associated economic and psychological burdens had detrimental impacts on individual wellbeing, especially for those being forced into quarantine [e.g., [1, 7–9]]. As one of the largest ethnic groups in Canada, Chinese migrants had faced unique and significant mental health challenges during the pandemic, including rising anti-Chinese discrimination, cultural and language barriers to access resources, and mental health stigma). Therefore, this interview study applies a life course approach to examine the psychological experiences and coping strategies among Chinese migrants in Canada, and its associated age and gender differences, during this unprecedented public health crisis [10].

### Psychological impacts of the pandemic

The psychological impacts of the pandemic and the associated predictors have been widely reported [e.g., [2]]. Women, young adults, and those with disabilities across a variety of racial groups were differentially more affected by the pandemic [e.g., [2, 3, 6, 11–14]].

#### Age differences

Research conducted in Western countries (e.g., U.S., Israel, Spain, and Sweden) generally revealed that when compared to their younger counterparts, older adults reported a relatively higher level of positive affect and resilience during the pandemic [15–20], a lower risk for pandemic-related stress, emotional distress, life change, and social isolation [13], and less pandemic-related social isolation and loneliness [21]. Similarly, older adults in the U.S. were less negatively impacted than young adults by perceived COVID-19 disruption. Furthermore, only young adults, not middle-aged or older adults, showed a positive relationship between perceived pandemic disruption and poor wellbeing [15]. A survey study also identified risk factors (e.g., concerns for others, uncertainty about the future, and fear of infection) and protective factors (e.g., faith, exercise/self-care, and nature) for psychological wellbeing among older adults in the U.S. during the pandemic [22].

#### Gender differences

With regards to the gender profile, a survey by Browning and colleagues [2] revealed a significantly higher level of psychological distress in women than men among non-Hispanic White, Black, Asian and Hispanic college students in the U.S.. Recent survey studies revealed the same gender difference among older Chinese immigrants in Canada [23]. Similarly, an online survey in Australia identified a differentially higher level of fear in middle-aged single women and mothers, presumably due to their financial insecurity related to potential job loss [24]. Finally, a study tracked the trajectories in daily wellbeing over an 11-week period in 2020 among Koreans and revealed a cubic pattern of fluctuation (i.e., initial decline, recovery, and substantial decline) that was more pronounced in younger adults and women than their counterparts [25]. Unlike other emotions, boredom was reported to be increased, while joy decreased in a linear fashion over time. This change had been differentially more evident among younger adults (relative to older adults) and men (relative to women) [25].

Taken together, aging showed a protective effect against the detrimental psychological impacts of the pandemic [13, 17, 25]. The pandemic elicited more negative psychological consequences in women than men [e.g., [2, 4, 25]]. However, little is known about age and gender profiles through an intersectionality lens [26]. A recent cross-sectional survey study sheds some light on this question by showing that in Spain, women experienced more physical symptoms, negative mood, and behavioral changes than men, irrespective of age [18]. However, in-depth studies with minoritized populations are scarce.

### Coping the adversity during the pandemic

Adaptive coping could effectively mitigate the detrimental psychological impacts of the pandemic. The use of adaptive coping strategies (e.g., approach-oriented) is correlated with positive emotional wellbeing in Western countries [27, 28]. Furthermore, resilience was associated with adaptive coping whereas stress was positively related to maladaptive coping [29, 30]. Coping has been categorized in different ways. In general, problem-oriented coping (e.g., problem-solving) was related to positive mental health outcomes, whereas emotion-focused coping (e.g., rumination, blame and avoidance) was linked to negative mental health outcomes [31, 32]. Additionally, approach-oriented coping promoted whereas avoidance-oriented coping disrupted psychological wellbeing of students in Spain [28].

Importantly, Garcini and his colleague [33] identified four types of coping strategies among Latinx communities in South Texas during the COVID-19 pandemic: behavioural, cognitive, social support and spirituality. Behaviour coping strategies include exercising, adjusting daily routines, taking public health precautionary measures; cognitive coping strategies include cognitive reappraisal, reframing attitude and outlook; and social coping strategies would include fostering social connection, seeking social support etc.

#### Age differences

Younger adults in the U.S. reported both adaptive (e.g., physical activity, cooking, developing routines, self-care and relaxation, social support) and maladaptive or avoidance coping (e.g., substance use or sleeping) during the pandemic [29]. On the other hand, older adults in the U.S. or U.K. were more likely to use adaptive and proactive coping strategies, such as exercising, modifying routines, adjusting attitudes, and staying socially connected [12, 34]. An interview with older adults in Midwestern U.S. showed a positive appraisal of coping and found that some emotion-focused coping strategies (e.g., cognitive avoidance) were also found to be adaptive during the early stages of the pandemic [17]. These results strengthened the counterintuitive perception of older adults as strong and resilient, instead of vulnerable, frail, weak, and disposable [12].

#### Gender differences

With regard to gender profiles, women were more likely than men to use a wide variety of coping strategies (e.g., emotional support) and react emotionally (e.g., feeling anxious, overwhelmed and stressed) in response to the COVID-19 crisis in the U.S. [34] and France [35]. Furthermore, female university students in Jordan expressed more distress-related symptoms and were more likely to cope by sleeping, studying, and worshiping relative to their male counterparts, who instead coped often by working, exercising, or playing video games [36]. In Canada, female university students were more likely than their male counterparts to use social media to cope with pandemic-related stress [37]. Using social media showed similar negative mental health effects for both genders, but substance use was associated with more negative impacts in men relative to women [37]. Pakistani students reported both emotion- and problem-based coping, but females differentially engaged in more social support, humanitarian (e.g., helping others) and acceptance strategies [38].

Taken together, past research suggested that adaptive coping strategies were effective in buffering the negative psychological outcomes [28, 31, 32]. Older adults were more adaptive in coping [12, 34], and women tended to engage in more coping than men [34, 35, 38]. The current study would take a step further to examine age and gender profiles in coping among Chinese migrants in Canada during the pandemic.

### Chinese migrants in Canada amidst the pandemic

Past research showed a disproportionately larger impact of the pandemic on racial and ethnic minority groups in the U.S. [39]. Specifically, non-Hispanic Black and Hispanic Americans showed greater concerns about the pandemic relative to White Americans [40]. Furthermore, pandemic-related mental health issues (e.g., depression rates) are disproportionately larger in racial/ethnic minority groups (e.g., Black, Hispanic, and Asian) in the U.S. and U.K. [41]. Canada is one of the most culturally diverse countries in the world. Chinese make up one of the largest ethnic groups in Canada, with 1.7 million people of Chinese ancestry accounting for nearly 4.7% of Canadian population [42].

A number of survey studies assessed psychological wellbeing and associated predictors of Chinese immigrants in Canada [e.g., [23, 43, 44]]. In a survey with 731 Mainland Chinese in Canada, 26.49% reported perceived and 10.40% reported experiencing anti-Chinese discrimination in April–May of 2020 [5]. In another survey, alarmingly over half (55%) of a sample of 471 Mainland Chinese in Canada reported perceived discrimination in May–June of 2020 [6]. In a third survey with 899 participants, 38.30% reported have perceived and 23.20% reported have experienced anti-Chinese discrimination in early 2021 [45]. Anti-Asian racism and discrimination was consistently reported as a robust predictor for poor psychological wellbeing in Chinese migrants in Canada [5, 6, 45–47]. A systematic review also showed that the rising anti-Asian stigma during the pandemic is detrimental to the psychological wellbeing of Asian Americans in the U.S. [48]. During the pandemic, Chinese immigrants developed a sense of “double unbelonging” due to the barriers of returning to China and their dissatisfaction with Western countries’ response to pandemic control in the early stage of the pandemic [49], which may magnify their psychological outcomes. Furthermore, the cultural identity confusion among second-generation Chinese immigrants may cause intergenerational conflicts in families and thus may pose a psychological wellbeing threat [50].

On the other hand, Chinese individuals are less likely to seek professional or social support for stress coping given the lack of culturally sensitive services as well as their collectivistic cultural background [e.g., [51]]. Specifically, older Chinese migrants often remained monolingual (e.g., speaking only Mandarin or Cantonese) and socialized primarily within the same ethnic groups [52]. The cultural/language barriers and mental health stigmas may further discourage them from seeking support [53, 54]. Therefore, it is important to understand the impacts of the pandemic on Chinese migrants’ psychological wellbeing and coping strategies during the pandemic.

### Theoretical perspectives

A few theoretical perspectives could explain the aforementioned age and gender differences. First, the *Socioemotional Selectivity Theory* [SST, [55, 56]] could explain the relatively lower negative impacts of the pandemic among older adults than younger ones [13, 15–21]. According to SST, with aging and a shrinking time horizon, older adults shift their goals to emotional gratification, thus they might be motivated to maintain emotional wellbeing and positivity. Furthermore, according to the *Strength and Vulnerability Integration* (SAVI) model [15], more vulnerable individuals might show higher strength/resilience to consequently engage in more adaptive coping strategies. This explains why adaptive coping was endorsed among older adults and women, given the heightened vulnerability for virus infection in older adults and greater life or emotional disruptions among women. Finally, according to the *Double Jeopardy Hypothesis* [57], the intersection of two negative status factors would cause a more detrimental impact than any one factor on its own. Considering that the pandemic has disproportionately larger impacts on younger adults, women, and racialized minority groups [4, 12], it is reasonable to predict that racialized and minoritized younger women would be most vulnerable to the impacts. This study aims to address this question with a semi-structured interview.

## The current study

Most of the aforementioned studies were conducted in Western countries and generally revealed more negative psychological experiences in younger adults relative to older ones [e.g., [12, 17, 37]] and in women relative to men [e.g., [2, 4]]. It was unclear whether the results could be generalized to Chinese individuals living in Canada who face unique challenges (e.g., discrimination, double-unbelonging) during the pandemic [e.g., [45, 49]]. Furthermore, none of the previous studies have used in-depth interviews to examine Chinese migrants’ psychological experiences of the pandemic. Compared to survey studies, qualitative interviews provide in-depth and open-ended information [58]. In this context, the current study aims to fill this gap to specifically examine pandemic-related emotional experiences and coping strategies of Chinese migrants in Canada with a semi-structured interview. Specifically, we target young (aged 18–39), middle-aged (aged 40–64), and old (aged 65 and over) non-Canada-born Chinese adults in Canada, including immigrants, students and visitors, a population that highly likely experienced a surge of racism during the pandemic [59].

In this context, this interview addresses the following research questions: 1) What are the common pandemic-related emotional experiences and coping strategies among non-Canada-born Chinese migrants in Canada during the pandemic? 2) Do older adults show more positive emotional experiences and engage in more adaptive coping strategies relative to younger adults? 3) Do women show more negative emotional experiences but engage in more adaptive coping strategies relative to men? In light of the *Double Jeopardy Hypothesis* [57], we predict that younger women might be most vulnerable to stressful emotional experiences and older women might be most adaptive in coping. As predicted by the SST [e.g., [55]], older adults might be least negative in emotions than other age groups. According to the SAVI [15], older adults and women would be more adaptive in coping compared to their counterpart groups.

## Methods

### Participants

Participants were recruited through an official post distributed through WeChat (a popular Chinese social media platform), the internet, and local Chinese communities through a Google registration link. By convention of a typical interview study, we aimed to recruit 20 adults (10 men and 10 women) from each of the three age groups: young (aged 18–39), middle-aged (aged 40–64), and old (aged 65 and above). A total of 120 participants signed up for the study. Only eligible participants were invited to participate, as per the following inclusion criteria: 1) within the target age range for each age group; 2) migrated from Mainland China; 3) have lived or plan to live in Canada for at least 6 months (including citizens, short-term visitors, permanent residents, and international students); and 4) can read and write Mandarin. The final sample included 20 young (age ranged 21–34, *M* = 28.11, *SD* = 3.82, 10 women and 10 men, 1 with missing age information), 21 middle-age (age ranged 42–57, *M* = 48.85, *SD* = 4.96, 11 women and 10 men, 1 with missing age information) and 20 old adults (age ranged 65–85, *M* = 73.45, *SD* = 6.30, 10 women and 10 men). One extra middle-aged woman was included due to a double-scheduling incidence. Table 1 displays the sample characteristics across age by gender groups. The sample was largely married/cohabited (62.3%), with a high level of education (75.4% with at least a university degree), and included citizens (34.4%), permanent residents (44.3%), international students (6.6%), and visitors with work/visit/travel permit (14.8%).

**Table 1:** Sample sociodemographic characteristics in frequency (percentage) within each group

Variable		Gender Group		Age Group^a^			Total
		Women (*n* = 31)	Men (*n* = 30)	Young (*n* = 20)	Middle (*n *= 21)	Older (*n* = 20)	
Marital Status	Single	6 (19%)	7 (23%)	11 (55%)	2 (10%)	0 (0%)	13 (21%)
	Widowed	3 (10%)	1 (3%)	0 (0%)	0 (0%)	4 (20%)	4 (7%)
	Divorced/Separated	6 (19%)	0 (0%)	1 (5%)	4 (19%)	1 (5%)	6 (10%)
	Married/Cohabited	16 (52%)	22 (73%)	8 (40%)	15 (71%)	15 (75%)	38 (62%)
Employment Status	Retired	11 (36%)	8 (27%)	0 (0%)	1 (5%)	18 (90%)	19 (31%)
	Student	4 (13%)	1 (3%)	5 (25%)	0 (0%)	0 (0%)	5 (8%)
	Unemployed	4 (13%)	1 (3%)	3 (15%)	2 (10%)	0 (0%)	5 (8%)
	Self-employed	2 (7%)	3 (10%)	0 (0%)	5 (24%)	0 (0%)	5 (8%)
	Contract/Temporary worker	2 (7%)	2 (7%)	1 (5%)	2 (10%)	1 (5%)	4 (7%)
	Full-time worker	8 (26%)	15 (50%)	11 (55%)	11 (52%)	1 (5%)	23 (38%)
Education	Middle School or below	3 (10%)	1 (3%)	0 (0%)	1 (5%)	3 (15%)	4 (7%)
	High School	4 (13%)	2 (7%)	0 (0%)	2 (10%)	4 (20%)	6 (10%)
	College	2 (7%)	3 (10%)	1 (5%)	2 (10%)	2 (10%)	5 (8%)
	University	12 (39%)	17 (57%)	13 (65%)	7 (33%)	9 (45%)	29 (48%)
	Master degree or above	10 (32%)	7 (23%)	6 (30%)	9 (43%)	2 (10%)	17 (28%)
Religion	None	22 (71%)	27 (90%)	17 (85%)	16 (76%)	16 (80%)	49 (80%)
	Missing/Both Christianism and Buddhism	1 (3%)	1 (3%)	1 (5%)	0 (0%)	1 (5%)	2 (3%)
	Christianism	8 (26%)	2 (7%)	2 (10%)	5 (24%)	3 (15%)	10 (16%)
Resident Status	International student	3 (10%)	1 (3%)	4 (20%)	0 (0%)	0 (0%)	4 (7%)
	Work permit/Visit/Travel	8 (26%)	1 (3%)	5 (25%)	3 (14%)	1 (5%)	9 (15%)
	Permanent resident	12 (39%)	15 (50%)	6 (30%)	6 (29%)	15 (75%)	27 (44%)
	Citizen	8 (26%)	13 (43%)	5 (25%)	12 (57%)	4 (20%)	21 (34%)
Family Income	Low	14 (47%)	14 (48%)	12 (60%)	9 (43%)	7 (39%)	28 (46%)
	High	16 (53%)	15 (52%)	8 (40%)	12 (57%)	11 (61%)	31 (51%)
Cultural Orientation	Neutral/No preference/Unsure	13 (43%)	12 (41%)	13 (68%)	7 (33%)	5 (26%)	25 (41%)
	East	7 (23%)	11 (38%)	4 (21%)	8 (38%)	6 (32%)	18 (30%)
	West	10 (33%)	6 (21%)	2 (11%)	6 (29%)	8 (42%)	16 (26%)
Self-rated health	Poor	12 (40%)	15 (52%)	11 (55%)	8 (38%)	8 (44%)	27 (44%)
	Good	18 (60%)	14 (48%)	9 (45%)	13 (62%)	10 (56%)	32 (52%)

^a^young (age range = 18–39), middled aged (age range = 40–64), and older (aged 65 +)

### Materials

The interview was conducted in Mandarin. The lead author developed an interview script for the research assistants (RAs) to follow during the interview (Appendix 1). The interview covers some demographic questions (e.g., age, gender, marital status, and length in Canada). These questions mainly check the inclusion eligibility and confirm the age/gender group category. To address the research questions, we interviewed participants on their daily life experiences (e.g., “How is your daily life affected by the pandemic?”), emotional experiences (e.g., “What are your major concerns/worries about the pandemic?”), and coping strategies (e.g., “What strategies have you used to regulate your fear and concerns?”). The current report specifically focused on the results from the emotional experiences and coping, as stratified by age and gender.

### Procedure

Recruitment took place from June 29th to September 2nd in 2020, when the Greater Toronto Area was in Stage 3 of the provincial reopening plan. The recruitment post included a link to the full consent form, a link to the Google registration form and its corresponding QR code. Based on the inclusion criteria and the “first come, first take” rule, only the eligible participants were contacted to be scheduled for the interview. Following the standardized interview procedure, all interviews were conducted through individual Zoom meetings or phone calls (Appendix 1). The consent form and the meeting information (e.g., Zoom ID or phone number confirmation) were sent to each participant the day before the scheduled appointment. An audio consent was collected at the beginning of the interview. The session was approximately one-hour long and audio-recorded. Participants subsequently received a $10 electronic gift card as compensation for their time and participation.

A total of nine RAs were involved in the operation of this project (see the acknowledgement section), primarily in data collection and coding. Three of them (co-authors) were deeply involved in final coding, data analysis, and manuscript preparation. To reduce potential expectation bias from the principal investigator, all the interview sessions and data coding were conducted by well-trained RAs who were blind to the specific research questions and hypotheses. Regular project meetings were held to tackle any data collection or coding issues and resolve any coding discrepancies to ensure methodological integrity.

### Coding and analysis approach

All the interview recordings were then transcribed verbatim and imported to NVivo for further coding and analysis. The demographic information collected during registration was linked with the interview files in NVivo. The data analysis procedure integrated both qualitative and quantitative approaches. Qualitative thematic analysis [60, 61] was used to identify and describe the major patterns (themes and subthemes) from participants’ responses. Thematic analysis is well-suited for investigating individual experiences and is widely used in health research [60]. Specifically, we followed a 4-stage coding approach. In Stage 1, the lead researchers read through all the transcripts thoroughly to extract memos and main points. In Stage 2, the same researchers discussed the memos and generated a preliminary master coding structure including codes and subcodes to categorize the content based on the major themes derived from the interview responses in light of the research questions. In Stage 3, the project lead (i.e., the lead author) introduced the master coding system to a team of RAs to follow in their coding of the interview transcripts. Two RAs independently coded each transcript file. In Stage 4, we gradually modified the master coding theme structure to capture mutually exclusive and distinct themes that could be clearly labeled and defined to ensure all RAs followed the same structure. Any discrepancies were first discussed and resolved between the two coders. Unresolved discrepancies were further discussed and resolved at weekly project meetings with the lead author. In Stage 5, we summarized, described, integrated, and quantitatively analyzed the results for this report. In addition, we also adopted content analysis [62] to identify the commonly used positive and negative emotional words.

Based on a thorough inspection of the interview responses, we first categorized responses to the emotional experience questions into positive and negative valence categories (in light of the SST) and then identified subthemes derived from specific narratives/expressions within each valence. Similarly, based on the initial inspection and overall assessment of the interview results, responses to the coping strategy questions were classified into behavioural, social, and cognitive coping themes, each further categorized into subthemes based on specific expressions within each theme.

For the supplementary quantitative analysis on the age and gender differences in emotional experiences and coping, we calculated the frequency of positive and negative emotional experiences, commonly used positive and negative emotional words, as well as the behavioural, social, and cognitive coping strategies reported by each participant (Table 2). The data were analyzed with mixed-design analysis of variance (ANOVA) models, each with age and gender as between-subjects variables and theme as a within-subject factor.

**Table 2:** Proportional unit frequency in each emotional experience, emotional words, and coping strategies across age and gender groups

		Women			Men			
		Young	Middle	Older	Young	Middle	Older	All
Emotional Experiences	Positive	0.60 (0.84)	0.82 (0.87)	1.20 (1.03)	0.40 (0.52)	1.00 (0.94)	1.20 (1.23)	0.87 (0.94)
	Self-oriented	0.00 (0.00)	0.27 (0.47)	0.20 (0.42)	0.10 (0.32)	0.30 (0.48)	0.30 (0.48)	0.20 (0.40)
	Other-oriented	0.20 (0.42)	0.45 (0.52)	0.60 0.70)	0.00 (0.00)	0.20 (0.42)	0.20 (0.42)	0.28 (0.49)
	Situation-oriented	0.40 (0.52)	0.09 (0.30)	0.40 (0.52)	0.30 (0.48)	0.50 (0.53)	0.70 (0.82)	0.39 (0.56)
	Negative	2.30 (0.95)	1.91 (1.04)	2.90 (1.37)	0.90 (0.88)	1.20 (0.79)	1.00 (0.82)	1.70 (1.20)
	Sadness	0.30 (0.48)	0.27 (0.65)	0.10 (0.32)	0.00 (0.00)	0.10 (0.32)	0.00 (0.00)	0.13 (0.39)
	Loneliness	0.40 (0.52)	0.18 (0.40)	0.70 (0.95)	0.20 (0.63)	0.00 (0.00)	0.40 (0.52)	0.31 (0.59)
	Fear	1.40 (0.70)	1.00 (0.63)	2.10 (0.74)	0.60 (0.52)	0.90 (0.74)	0.50 (0.53)	1.08 (0.82)
	Anger	0.20 (0.42)	0.45 (0.52)	0.00 (0.00)	0.10 (0.32)	0.20 (0.42)	0.10 (0.32)	0.18 (0.39)
Emotional Words	Positive words	0.60 (1.26)	1.55 (3.05)	1.30 (1.89)	1.20 (2.57)	1.20 (1.81)	1.60 (1.65)	1.25 (2.08)
	Happiness	0.50 (0.97)	0.73 (2.41)	1.00 (1.70)	0.40 (0.70)	0.10 (0.32)	0.50 (1.27)	0.54 (1.40)
	Optimism/confidence	0.10 (0.32)	0.82 (1.25)	0.30 (0.67)	0.80 (2.20)	1.10 (1.60)	1.10 (1.20)	0.71 (1.35)
	Negative words	8.80 (4.78)	9.36 (5.03)	7.20 (5.75)	3.70 (3.83)	5.50 (5.38)	2.70 (3.56)	6.26 (5.23)
	Sadness	0.60 (1.26)	0.82 (2.71)	1.40 (3.50)	0.10 (0.32)	0.10 (0.32)	0.00 (0.00)	0.51 (1.89)
	Anxiety	2.20 (2.82)	2.00 (2.57)	0.30 (0.48)	0.60 (1.26)	0.20 (0.42)	1.10 (3.48)	1.08 (2.25)
	Worry	3.70 (2.36)	4.36 (3.80)	1.60 (1.96)	2.30 (2.54)	4.50 (4.86)	1.20 (1.48)	2.97 (3.22)
	Fear	2.20 (2.44)	1.09 (1.51)	3.80 (4.16)	0.70 (0.82)	0.60 (1.35)	0.40 (1.26)	1.46 (2.43)
	Anger	0.10 (0.32)	1.09 (1.22)	0.10 (0.32)	0.00 (0.00)	0.10 (0.32)	0.00 (0.00)	0.25 (0.67)
Coping Strategies	Behavioural	1.10 (1.37)	0.91 (1.22)	2.60 (1.35)	0.20 (0.42)	1.50 (1.35)	1.30 (1.57)	1.26 (1.41)
	Personal growth	0.60 (0.97)	0.27 (0.47)	0.40 (0.52)	0.00 (0.00)	0.90 (0.99)	0.40 (0.70)	0.43 (0.72)
	Physical exercise	0.20 (0.42)	0.09 (0.30)	0.30 (0.48)	0.00 (0.00)	0.40 (0.52)	0.30 (0.48)	0.21 (0.41)
	Relaxation	0.10 (0.32)	0.09 (0.30)	0.20 (0.42)	0.00 (0.00)	0.10 (0.32)	0.00 (0.00)	0.08 (0.28)
	Preventive measures	0.20 (0.42)	0.45 (0.82)	1.60 (0.84)	0.10 (0.32)	0.10 (0.32)	0.50 (0.85)	0.49 (0.81)
	Humor	0.00 (0.00)	0.00 (0.00)	0.10 (0.32)	0.10 (0.32)	0.00 (0.00)	0.10 (0.32)	0.05 (0.22)
	Social	0.50 (0.71)	0.36 (0.50)	1.10 (1.20)	0.10 (0.32)	0.60 (0.97)	0.40 (0.70)	0.51 (0.81)
	Family bonding	0.10 (0.32)	0.00 (0.00)	0.10 (0.32)	0.10 (0.32)	0.40 (0.70)	0.10 (0.32)	0.13 (0.39)
	Socialization	0.30 (0.48)	0.27 (0.47)	0.30 (0.48)	0.00 (0.00)	0.20 (0.42)	0.10 (0.32)	0.20 (0.40)
	Social support	0.00 (0.00)	0.09 (0.30)	0.60 (0.84)	0.00 (0.00)	0.00 (0.00)	0.20 (0.42)	0.15 (0.44)
	Volunteering	0.10 (0.32)	0.00 (0.00)	0.10 (0.32)	0.00 (0.00)	0.00 (0.00)	0.00 (0.00)	0.03 (0.18)
	Cognitive	0.00 (0.00)	0.55 (0.69)	0.10 (0.32)	0.00 (0.00)	0.20 (0.42)	0.00 (0.00)	0.15 (0.40)

## Results

We reported the results in two major sections: emotional experiences towards the pandemic (emotional experiences and emotional word use) and coping. In each section or subsection, we reported qualitative thematic analysis descriptions first, followed by the supplementary quantitative analyses on frequency data. The main purpose of the supplementary quantitative analyses was to provide preliminary descriptive statistics to further support and verify the primary result patterns emerged in the qualitative analysis. However, please note that given the small sample size and the non-random sampling approach, some quantitative analyses might lack sufficient statistical power. We would refrain from drawing firm conclusions merely based on quantitative analyses and be cautious in the interpretation of these results.

### Pandemic-related emotional experiences

#### Emotional experiences

Guided by the SST, our primary focus is on the positive over negative response bias. Therefore, the thematic analysis using NVivo extracted two broad themes from the responses to the psychological impact question: positive and negative emotional experiences, with some subthemes identified within each (see Table 2).

*Positive emotional experiences*. Positive emotional experiences refer to pandemic experiences perceived as positive in emotional valence. This theme included three subthemes: self-oriented, other-oriented, and situation-oriented positive emotions.

*Self-oriented positive emotions*. These emotions describe emotional experiences towards themselves (e.g., content, joyful, faithful, proud, confident, powerful, courageous). For example, some middle-aged women reported joyful/faithful feelings by taking a positive perspective (e.g., “How can I live my life if I am not happy everyday? As a matter of fact, I am very happy. Although many bad things are happening in the world, you can still think there are also many things that make you feel joyful”) or through religion/faith (e.g., “I am a Christian, so I rely on God instead of the environment. I feel joyful and peaceful in Him”). An old woman felt content and fulfilled about life because having things to do during the pandemic (“With the passage of time, I felt much better. There is something I can do everyday, then I feel that life is becoming more fulfilled”). Some middle-aged men expressed courage facing the pandemic [“I have never been scared of this (pandemic)”] and pride in their Chinese identity and Chinese medicine in the management of COVID-19 virus spread (“I am proud of myself for being a Chinese, for Chinese culture and Chinese medicine”).

*Other-oriented positive emotions*. These emotions describe emotional experiences related to interactions with others (e.g., secure, loving, thankful). A middle-aged woman expressed a sense of security towards the Canadian government’s effort in combating discrimination: “I think that the Canadian government is more supportive of the effort to combat discrimination and this makes me feel safer compared to the United States”. Middle-aged and old women expressed a greater sense of calm and peace especially by staying home during the lockdown. For example, a middle-aged woman stated “I do not have much emotional fluctuation, and generally felt calm and peaceful because I stayed home most of the time…”. Moreover, an older woman said “it is also beneficial to pace down and stay at home, with more space, more time, and more freedom. I felt that everything became quiet and peaceful”. Furthermore, a middle-aged woman expressed appreciation and thankfulness: “Western people treat us friendly with warm smiles and kind greetings, I feel good about this” and an old man expressed appreciation/thankfulness towards his neighbour: “My neighbour mowed my front yard lawn without even being asked and my wife let me express our appreciation”.

*Situation-oriented positive emotions*. These emotions (e.g., optimistic, hopeful, trusting, accepting) describe emotional experiences related to the situation/environment associated with the pandemic. Some old adults felt optimistic and hopeful about the pandemic control, such as “I felt that the government really cares about people and serves as a strong support during the pandemic, this makes us feel hopeful and grateful” and “I am hopeful and thankful that one day we will have effective vaccination and we will feel safe to go out or even go back to China”. Furthermore, an old man expressed his optimism about the pandemic control: “I am certain that we will get the pandemic under control, I am optimistic about the future” and another old man described his effort to be optimistic: “try to live a happy life in a different way, try not to be pessimistic”. Some young adults expressed trust and acceptance for changes in life due to the pandemic. For example, a young man described an accepting attitude “I can accept many changes now” and another young man reported that “we know how to prevent the virus, and we know that sometimes it would be impossible for everything to be fully under our control”.

Table 2 displays the frequency data in each subtheme of positive emotional experiences. The 3 (age) × 2 (gender) × 3 (subtheme) ANOVA revealed a significant main effect of subtheme, *F*(2, 110) = 3.28, *p* = 0.041, *ηp*^*2*^ = 0.06, qualified by a subtheme by gender interaction, *F*(2, 110) = 5.03, *p* = 0.008, *ηp*^*2*^ = 0.08. All the other effects were not significant (*p*s ≥ 0.06). Follow-up pairwise comparisons (with Bonferroni correction) showed more other-oriented positive experiences reported by women than men (*p* = 0.020), but no gender differences in self-oriented or situation-oriented emotions (*p*s ≥ 0.15). On the other hand, women reported more other-oriented than self-oriented emotions (*p* = 0.045) whereas men reported more situation-oriented than other-oriented emotions (*p* = 0.008). Overall, women experienced more other-oriented positive emotions than men. On the other hand, women experienced more other-oriented than self-oriented positive emotions whereas men experienced more situation-oriented than other-oriented emotions. This may reflect the unique need or expectation for social roles, socialization and caring among women as per gender-related social stereotypes.

*Negative emotional experiences*. Negative experiences were related to experiences with a negative emotional valence and can be further captured in four subthemes: sadness, loneliness, fear, and anger.

Sadness (depressed, despair, hopeless). Some young women expressed a feeling of hopelessness due to the prolonged length of the pandemic (i.e., “I feel that there is no end to the pandemic and it feels hopeless”), a feeling of sadness/distress because of job loss (i.e., “I think I'm pretty pessimistic…I must have been sad for a long time because I lost my job…so it was definitely a time of sadness”) or uncertainty about the future (i.e., “I am uncertain what it will be like for the future… but the pandemic made me unhappy and distressed or even depressed”). A middle-aged woman described her sadness towards pandemic-related restrictions as “Pandemic made it impossible for me to go back [to China] to visit my mom, who is in poor health, and I felt really down about this”.

*Loneliness (isolated, socially disconnected)*. Some older women expressed severe loneliness due to the stay-at-home order and lack of friends in Canada (e.g., “The lifestyle has changed because I can only stay in my house or backyard… I feel lonely and helpless”; “I have no friends here, so I feel very lonely, very lonely here”). A young man expressed a feeling of social disconnection during the lockdown: “I feel more lonely (than before) because of the reduced social interaction and friends”.

*Fear (scared, anxious, worried, insecure, nervous, threatened)*. Fear is the most prevalent and commonly reported negative emotional experience during the pandemic. For example, a middle-aged woman stated concerns and worries for close others: “I am very anxious and stressed, because my husband works in a hospital and my daughter is a pharmacist”. An old woman expressed her catastrophic contraction fear and worry as “I am scared. The whole family will get infected if anyone in the family gets infected. This will spread to our neighbors, the community, and the town. This is most frightening”.

*Anger (irritable, furious, and hateful)*. The sample reported anger and irritation towards the pandemic and related risks and restrictions. For example, a middle-aged woman expressed her anger and irritability over the likelihood of being discriminated against and children’s disobedient behavior (i.e., “I feel irritable knowing that if I go back to China, I might face discrimination upon coming back. To make it worse, some minor things might trigger my anger, such as when my kids do not listen to me”). Furthermore, they also expressed furious feelings and anger towards the government’s slow actions in pandemic control and anti-Chinese discrimination media reports (i.e., “I feel furious about the slow action of the public health offices and anti-Chinese discrimination reports in medias” by a middle-aged woman) and the misinformation in news (i.e., “Very angry at the news from China” by a middle-aged man). A middle-aged man expressed his irritation as “I surely have some negative emotions, like a bit irritated” and a young woman expressed her resentfulness towards the unexpected life disruption as “I missed my husband who is currently in China. I resented how the pandemic disrupted my life which led to many negative thoughts”.

The quantitative frequency data in each subtheme of negative emotional experiences were displayed in Table 2. The 3 (age) × 2 (gender) × 4 (subtheme) ANOVA revealed a significant main effect of subtheme, *F*(3,165) = 45.94, *p* < 0.001, *ηp*^*2*^ = 0.46, qualified by a subtheme by gender interaction, *F*(3,165) = 6.62, *p* < 0.001, *ηp*^*2*^ = 0.11; a subtheme by age interaction, *F*(3,165) = 2.63, *p* = 0.019, *ηp*^*2*^ = 0.09. Women reported significantly more fear than men (*p* < 0.001), but there were no significant gender differences in other negative emotions (*p*s ≥ 0.057). Young and middle-aged group reported more fear than other negative experiences (*p*s ≤ 0.007), whereas older adults reported more fear and loneliness than other negative experiences (*p*s ≤ 0.018). The 3-way interaction was also significant, *F*(3,165) = 3.36, *p* = 0.004, *ηp*^*2*^ = 0.11. The follow-up 3 (age) by 2 (gender) ANOVA on each emotion revealed a significant age by gender interaction only in fear, *F*(2,55) = 6.86, *p* = 0.002, *ηp*^*2*^ = 0.20. Women reported more fear than men, significant in old and young (*p*s ≤ 0.008), but not middle-aged group (*p* = 0.725). For women, old adults reported more fear experiences than middle-aged adults (*p* = 0.001). No age differences were identified for men (*p*s ≥ 0.519). Older adults also experienced differentially more loneliness. Women, particularly older women, are more vulnerable to fear experiences.

**Fig. 1:**
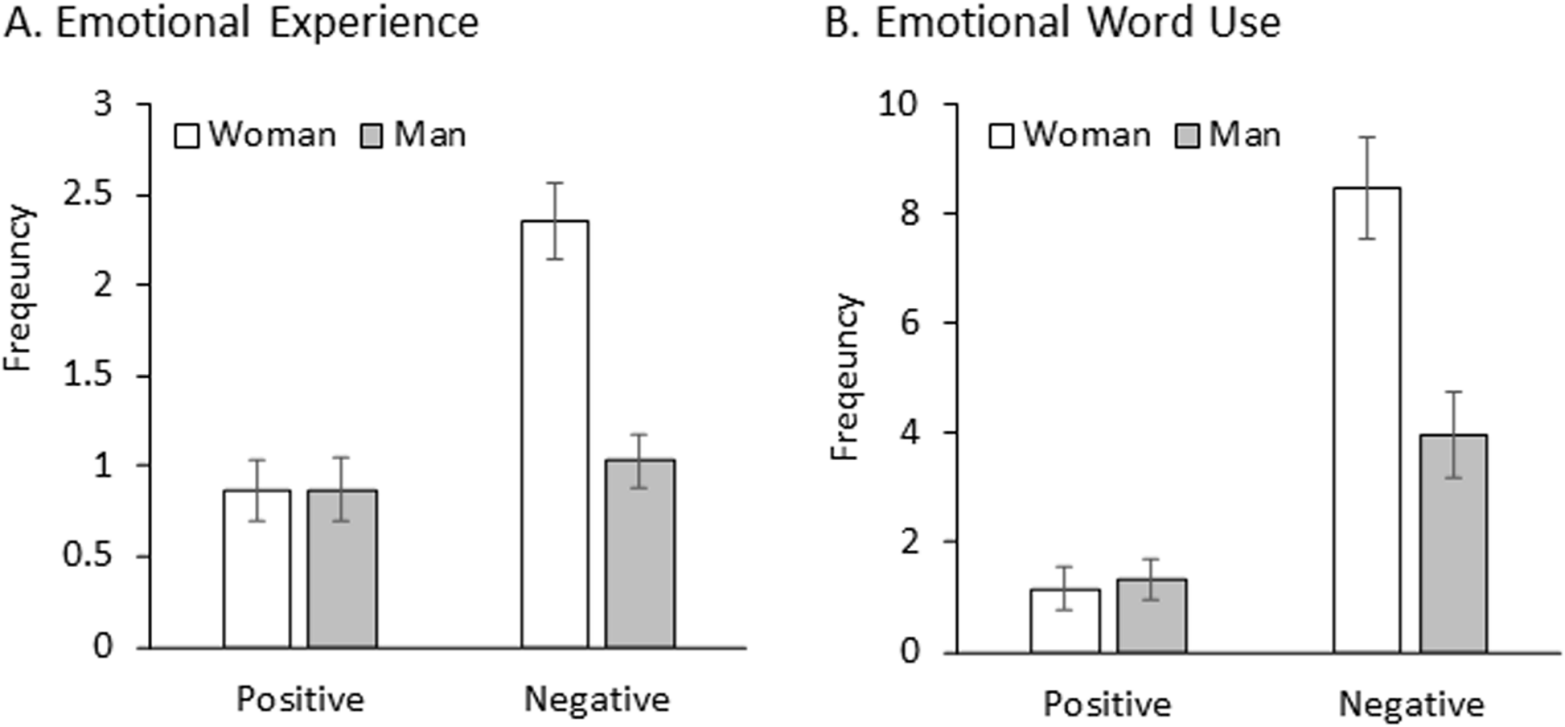
Frequency of positive and negative emotional experiences and emotional words used by woman and man participants

Taken together, a 3 (age) × 2 (gender) × 2 (valence) ANVOA revealed main effects of valence [*F*(1,55) = 20.55, *p* < 0.001, *ηp*^*2*^ = 0.27], age [*F*(2,55) = 3.43, *p* = 0.039, *ηp*^*2*^ = 0.11], and gender [*F*(1,55) = 16.54,, *p* < 0.001, *ηp*^*2*^ = 0.23]. Negative experiences were more prevalent than positive experiences, older adults reported more emotional experiences than young adults, and women reported more emotional experiences than men. The valence by gender interaction was significant, *F*(1,55) = 13.41, *p* = 0.001, *ηp*^*2*^ = 0.19. The valence effect was significant only in women (*p* < 0.001) but not men (*p* = 0.527). All the other effects were not significant (*p*s ≥ 0.208). Overall, the pandemic emotional experience was predominantly negative, particularly among women. The results were depicted in Panel A of Fig. 1.

#### Emotional word use

As a supplementary analysis to further verify the emotional experience result patterns, the content analysis using NVivo identified commonly used positive and negative words from the participants’ responses to the psychological/emotional impact questions (Table 2). Most commonly used positive words included words related to the feelings of *happiness* (“开心” or “愉快” in Chinese) and power/strength-related *optimism/confidence* (“信心”, “乐观”, or “积极” in Chinese), and most commonly used negative words included words related to the feelings of *worry* (“担心”), *fear* (“害怕”), *anxiety* (“焦虑”), *sadness* (“难过” or “伤心”), and *anger* (“生气” or “愤怒”).

Table 2 presents the aggregated frequency (number of occurrences) of these identified words during the interviews. The Text Search query function in NVivo was used to extract the specific words, stratified by age and gender groups. All repetitions resulted from self-reiterations that happened naturally during the interviews, words that describe others’ emotions, used by the interviewer, or implying lack of certain emotions (e.g., “not worried”) were excluded from the calculations. Therefore, the data displayed in Table 2 denotes the average frequency of each specific word used in the interview across age by gender groups.

##### Positive words

The analysis on positive word use frequency data revealed a gender by theme interaction, *F*(1,55) = 4.89, *p* = 0.031, *ηp*^*2*^ = 0.08. All the other effects were not significant (*p*s ≥ 0.469). The follow-up pairwise comparisons showed that men used more power/strength-related words (i.e., optimism, confidence, positive) than words describing happiness (*p* = 0.044) whereas women did not differ between the two (*p* = 0.295).

##### Negative words

The analysis on negative word use frequency data revealed significant main effects of theme [*F*(4,220) = 14.95, *p* < 0.001, *ηp*^*2*^ = 0.21] and gender [*F*(1,55) = 13.37, *p* = 0.001, *ηp*^*2*^ = 0.20]. Women used more negative words relative to men. “Worry” was most frequently used than any other negative words (*p*s ≤ 0.022), and “fear” or “anxiety” were used more often than “anger” (*p*s ≤ 0.027). The theme by age interaction was also significant, *F*(8,220) = 2.91, *p* = 0.004, *ηp*^*2*^ = 0.10. The pairwise comparisons showed that young adults used “worry” more frequently than “sadness” or “anger” (*p*s ≤ 0.024), middle-aged used “worry” more frequently than any other negative words (*p*s ≤ 0.001), and older adults used more “fear” than “anger” (*p* = 0.003). Taken together, these results suggested women used more negative words than men and that “worry” was the most frequently used word, particularly by middle-aged and young adults.

Taken together, a 3 (age) × 2 (gender) × 2 (valence) ANOVA on word use frequency revealed main effects of valence [*F*(1,55) = 65.49, *p* < 0.001, *ηp*^*2*^ = 0.54] and gender [*F*(1,55) = 8.78, *p* = 0.004, *ηp*^*2*^ = 0.14], qualified by an valence by gender interaction, *F*(1,55) = 14.48, *p* < 0.001, *ηp*^*2*^ = 0.21. Pairwise comparisons showed no gender effect in positive word use (*p* = 0.738), but women used negative words more frequently than men (*p* = 0.001). On the other hand, the valence effect was significant in both gender groups (*p*s ≤ 0.004), but the mean difference between the two valence categories was larger in women (7.306) than men (2.633). The pattern was largely consistent with the emotional experience analysis, suggesting that women experience more negative emotions than men during the pandemic. The results were depicted in the Panel B of Fig. 1.

#### Coping strategies during the pandemic

Three types of coping strategies were derived based on participants’ specific responses: behavioural, social, and cognitive strategies. The classification largely aligns with those of Garcini et al. [33], except for the absence of spirituality-related strategies. These strategies were largely described as effective and adaptive by participants.

##### Behavioural coping

Behavioural coping refers to the active use of adaptive behaviours or actions to cope with adversity, including personal growth (e.g., learning, reading, and hobbies), physical exercise, relaxation (e.g., listening to music, practicing Tai Chi and Yoga), preventive measures (e.g., using personal protective equipment, keeping social distance, and following public health measures), and humour (Table 2).

Personal growth activities were commonly used as a behavioural coping strategy. Some women reported engaging in activities related to personal growth, such as taking courses and reading (e.g., “I can spend more time on important things, such as taking some online courses” by a young woman; “I attended a CBT class last year and thought it was very helpful. Now I am reading related books and sharing information with my kids on emotional regulation” by a middle-aged woman). An old man reported community learning activities (i.e., “The community offered us programs to learn English online. I also joined a choir to sing online”). A middle-aged man developed a new hobby, fishing. (i.e., “Now I like fishing. My mood would improve when I go fishing”).

Participants also reported physical exercise and relaxation strategies. For example, an old man engaged in indoor exercise during the lockdown (i.e., “I do home-based indoor exercise when we were not allowed to go out to walk or jog”), an old woman reported “we usually have a morning walk outside”, and a middle-aged woman reported a relaxation strategy as “It was very effective to calm myself by driving to the countryside and enjoying the nature”.

Preventive measures were also commonly reported, especially by women and older adults. For example, a middle-aged woman reported a sense of security by wearing a mask (i.e., “I wear a mask whenever I go out, and I feel safe in Chinese supermarkets that required wearing a mask”) and some old women reported adherence of behavioral measures (e.g., “we keep distance from others, sometimes with masks on and we wash our hands right after we come back [from a morning walk]” by an older woman, and “sometimes, if I wash my hands for less than 20 s, I will redo it. I will rinse my mouth with salty water before drinking water when returning from outside” by another older woman). An older woman used humor to cope (i.e., “sometimes I will share jokes and funny games on WeChat”).

##### Social coping

Social coping refers to the endorsement of activities in social networking, connection, and support. It includes family bonding (e.g., spending more time with family, planning family activities), socialization (e.g., online and in-person social interactions), receiving social support (e.g., family, work, and community support), and volunteering. For example, some middle-aged men reported family bonding by spending time with family (e.g., kids) to engage in a variety of activities such as boating, kayaking, fishing, biking, and board games (e.g., “Play with kids in the lake like boating, kayaking, …and fishing” by a middle-aged man, and “Spending more time with family, biking with kids…, playing MaJiang with older adults” by another middle-aged man).

Participants also reported engagement of in-person socialization (e.g., “we seniors were chatting in a community activity room while the distance between chairs were being marked to keep social distance” by an older woman, and “I called my friends to go fishing in a remote place where there was no one else” by a middle-aged man) and online socialization (e.g., “when I felt lonely and helpless, I would video chat with my friends and tell them my feelings” by a young woman, and “[by attending] Zoom meetings and online social activities such as performing, poetry reading and skill training workshops, you can maintain a positive mood during the quarantine” by a middle-aged woman) during the quarantine. Some older adults reported receiving social support. For example, an old man reported that “for the first month of the pandemic, I asked my children to deliver food”.

Volunteering experience was also reported as a behavioural coping strategy. For example, a young woman reported “then I learned something that I didn’t have time to learn before…and then I did volunteer”. An old woman reported “I took on a volunteer position, working from 4 to 5 pm every day. I will start my shift in around 3 min after this interview. During my 1-h shift, I am on duty for an on-line community platform…[by participating in] a variety of community volunteer opportunities, my panic and anxiety towards the pandemic is… reduced”.

##### Cognitive coping

Cognitive coping refers to the adjustment of perception, belief or thinking, such as dividing attention, avoiding negative news, reappraisal, and adjusting expectations. For example, a middle-aged man reported dividing attention to cope “by focusing on work and being prepared for the future, I feel I can regulate my negative emotions.” Some middle-aged women tried to avoid negative news about the pandemic (e.g., “We intentionally avoid this type of news. I do not even look at such reports as the daily increase of cases in the U.S. and Canada. I tried not to look at and even consciously avoided negative things [about the pandemic]”). A middle-aged woman described cognitive reappraisal strategy as “we better pay attention to the positive side of it (pandemic)” and another middle-aged woman reappraised her complaints about her husband playing games “I stopped complaining because I can understand that it is really boring and he can’t go out, what else can he do?”. Individuals also adjust their expectations to cope. For example, a middle-aged woman adjusted her expectation from thriving to surviving “Instead of thriving, we aim for surviving…I do not feel much pressure”, and a middle-aged man adjust his expectation by being prepared to downgrade housing “if no new client due to the instability of the pandemic, I may not be able to afford a [big] house, and then I will need to downgrade to a smaller house or even rental”.

**Fig. 2:**
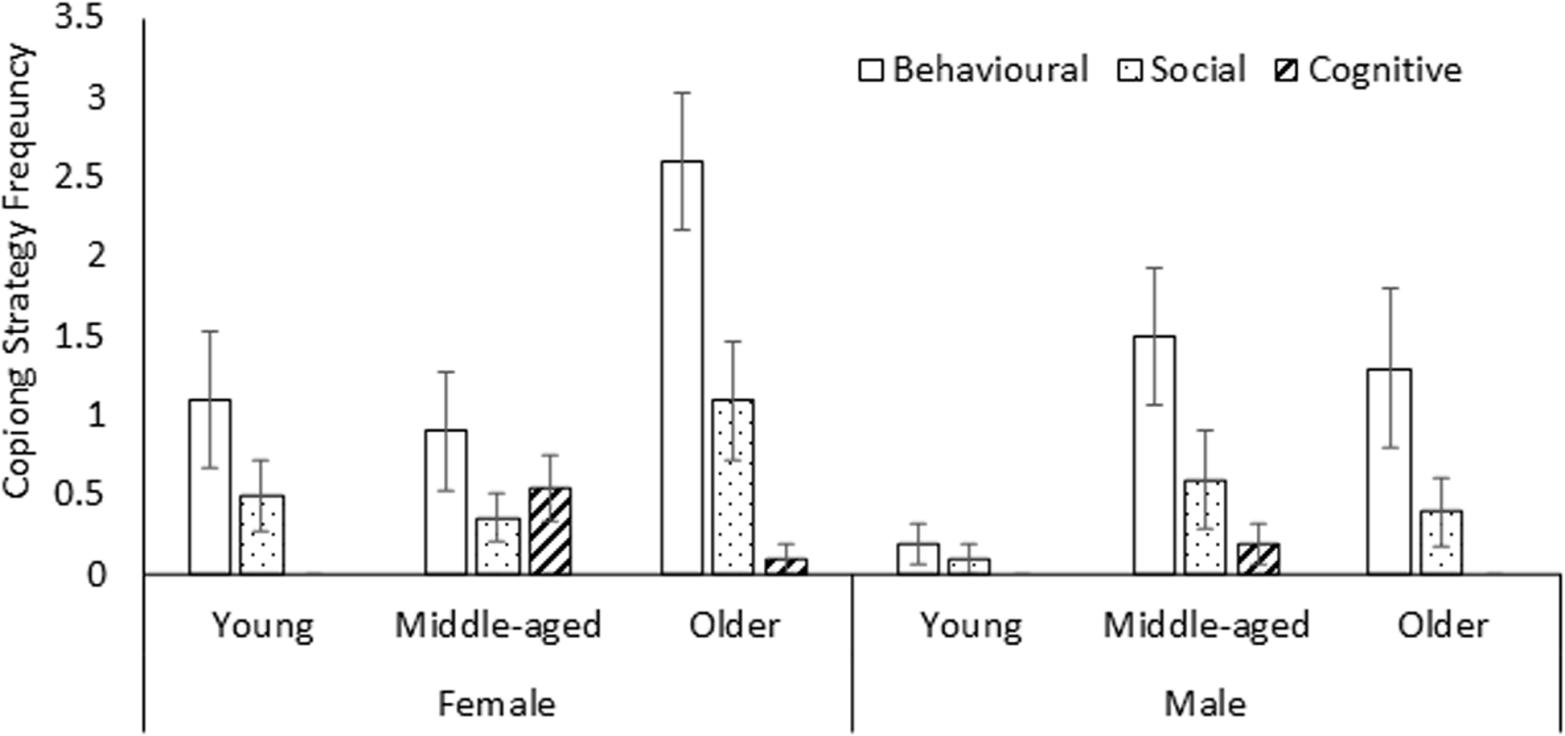
Frequency of behavioural, social, and cognitive coping strategies stratified by age and gender groups

The quantitative coping frequency data were presented in Table 2. The 3 (age) × 2 (gender) × 3 (coping theme) ANOVA revealed significant main effects of coping theme [*F*(2,110) = 30.75, *p* < 0.001, *ηp*^*2*^ = 0.36], age [*F*(2,55) = 5.28, *p* = 0.008, *ηp*^*2*^ = 0.16], and gender [*F*(2,55) = 4.61, *p* = 0.036, *ηp*^*2*^ = 0.08]. Participants reported more frequent use of behavioural than social, followed by cognitive strategies (*ps* ≤ 0.006). Women reported more coping than men, and older adults reported more coping than young adults (*ps* ≤ 0.036). There was a theme by age interaction, *F*(2,110) = 3.73, *p* = 0.007, *ηp*^*2*^ = 0.12, qualified by a 3-way interaction, *F*(2,110) = 2.64, *p* = 0.037, *ηp*^*2*^ = 0.08. Follow-up analyses revealed a theme by age interaction only for women (*p* = 0.004), but not men (*p* = 0.125). Pairwise comparisons showed that older adults reported more frequent behavioral coping than middle-aged or young women (*ps* ≤ 0.049), middle-aged women reported more frequent cognitive coping than young women (*p* = 0.028). All the other comparisons were not significant (*p*s ≥ 0.092). Taken together, behavioural strategies were reported most frequently, particularly by older women. These results were displayed in Fig. 2.

## Discussion

Our study assessed the emotional experiences and coping strategies among non-Canada-born Chinese migrants in Canada during the pandemic. The results revealed some common themes in emotional experience and coping strategies, as well as some preliminary associated age and gender profiles in these themes.

### Emotional experiences

The results identified some positive experiences, including an optimistic and trusting attitude towards the pandemic situation and related measures, a sense of security, peace, or gratitude towards others during the lockdown, and some positive self-oriented feelings, such as being content, proud, confident, and courageous despite adversity. These positive experiences demonstrate psychological resilience and strength of this sample. Furthermore, the analysis also identified some frequently used positive words implying happiness (e.g., happy, joy) or some power- or strength-related words (e.g., optimistic, confident, active).

Nevertheless, the results suggested that pandemic emotional experience is overwhelmingly negative. Commonly experienced negative emotional experiences during the pandemic included fear, loneliness, sadness, and anger. Fear was most prevalently reported, particularly in women. Older adults also showed a differentially higher level of loneliness. Additionally, the analysis on commonly used emotional words identified some frequently used negative words with a meaning of fear, anxiety, worry, sadness and anger. The overwhelmingly negative emotional experiences might be related to the uncertainty of the pandemic, rising psychological stress and risk factors experienced by Chinese Canadians, such as anti-Asian racism and discrimination [46] and a sense of “double-unbelonging” [49], stigmas related to mental illnesses [49], and barriers related to seeking services and support [53, 54] among this minoritized population.

#### Age profile

Little age difference was detected in positive emotional experiences or positive word use. These results are generally inconsistent with an earlier finding that older adults still hold an optimistic view and expressed more positive emotions than younger adults during the pandemic [16, 20], and the results also failed to support the SST [55, 56]. This is possibly because older Chinese migrants in Canada may face a variety of exceptional barriers (e.g., language/cultural/social barriers) and challenges (e.g., rising discrimination, medical care accessibility) during the pandemic. These challenges may have masked the positivity effect in their emotional experiences. Nevertheless, it should be noted that despite their physical vulnerability, social adversity, and exceptional language/cultural/social barriers, older adults did not experience more negative emotions than other age groups, suggesting a maintained psychological strength/resilience among older adults during the pandemic, which partially supports the SAVI model [15].

For negative emotional experiences, the results suggested that older women reported a proportionally higher level of fear towards the pandemic relative to the other age and gender groups. In addition, older adults also reported a differentially higher level of fear, followed by loneliness relative to other negative emotions. The heightened fear and loneliness in older adults are likely due to their increased social disconnection during the lockdown period given their high vulnerability to virus infection relative to other age groups. Considering women are likely to be emotional or practical caregivers in the family and society and they may focus more on family and social relationship and care more about others’ health conditions, the pandemic-related behavioural measures (e.g., lockdown, school closure etc.) may add an extra toll on their life routines and emotional conditions. However, older adults may lack sufficient resources to effectively adapt to all these changes. As a result, older women experienced some exceptional negative emotions, fear specifically, towards the pandemic. Furthermore, the lockdown measures may have further restricted older adults’ physical and social activities, making it risky for them to visit friends and family (e.g., their children or grandchildren). Given the heightened infection risk for older adults, they may be particularly more impacted by the social distancing and restrictions at the early stage of the pandemic. This would contribute to their heightened loneliness.

In the analysis of emotional word use “worry” was found to be the most frequently used negative word, especially among middle-aged and young adults. Notably, young adults may have experienced the most dramatic changes in their daily life (e.g., online schooling, remote work, and limited socialization) during the pandemic. These changes added to the stressors commonly experienced by students or graduates, such as maintaining academic performance, securing employment, and building social networking etc. However, they are probably not mature or independent enough yet to effectively regulate their negative emotions [36]. Additionally, young adults’ time spent on social media might also have elicited social comparisons and related anxiety and worry about self-images [36, 63]. Furthermore, the middle-aged adults might be more disrupted in career/job/family during the pandemic, given their dominant role in all these areas, and this may heighten their uncertainty and worry about the impacts of the pandemic on their personal life and the society’s future.

#### Gender profile

With regards to the gender profile in positive emotional experience, women generally experienced more other-oriented than self-oriented positive emotions whereas men experienced more situation-oriented than other-oriented emotions. Interestingly, power/strength-related positive words were more commonly used by men than women. Given the gender stereotype in social and family expectations, especially in traditional Chinese culture, women typically take on more caregiving responsibilities (e.g., child-care, family support). As a result, positive emotions are predominantly other-oriented in women, but more situational in men. This result is largely consistent with a study showing more humanitarian engagement (e.g., helping others) in women than men among Pakistani students [38]. Interestingly, men used more power/strength-related positive words (e.g., optimism or confidence), possibly reflecting the well-documented stereotypical gender differences in psychological needs, with women prioritizing connection and relationship while men prioritize sense of value and ability [64].

On the overall gender profiles in negative emotions, the pandemic emotional experience and emotional word use were predominantly negative, especially in women than men, regardless of age. The result is consistent with earlier finding of age-equivalent gender differences in negative mood [18]. Consistent with literature [2, 4, 11, 12, 25], the current study showed that women’s emotional states were more negatively impacted by the pandemic, possibly because the changes in schooling and socialization from the pandemic caused more disruptions in women’ daily activities, work schedule, and work/family balance [24].

### Coping strategies

Largely consistent with the coping strategy classification in Garcini et al. [33], this interview identified a variety of coping strategies falling under behavioural, social, and cognitive coping categories, in an order of prevalence. Although a recent work showed social and alcohol coping were maladaptive whereas resilience and relaxation were adaptive for mental health during the pandemic in the U.S. [20], almost all the reported coping strategies in the current interview were described as adaptive and effective by participants. Past research also showed that even emotion-focused coping strategies (e.g., cognitive avoidance) were adaptive in early pandemic [17]. Considering that adaptive coping can reduce negative psychological outcomes [32] and boost resilience and emotional wellbeing in Western countries [27, 29, 30], we strongly believe that these coping strategies were important for Chinese migrants to remain resilient during the pandemic.

#### Age profile

The results showed that in general older adults reported engaging in more coping behaviors relative to the young adults. Specifically, older women engaged in more behavioral strategies relative to both middle-aged and young women, and middle-aged women engaged in more cognitive strategies than young women. These findings confirmed the high level of resilience and adaptability among older adults during the pandemic [12, 13, 20, 34]. In light of the SST [55, 56], with a tightened time horizon (perceiving time left as more limited), older adults are more likely to pursue emotionally fulfilling goals and prioritize emotional wellbeing over knowledge/information acquisition. Directed by this goal shift, older adults are more motivated to actively and adaptively cope with adversity with available resources. The finding adds to literature and further strengthened the counterintuitive perception of older adults as resilient despite their physical vulnerability during the pandemic [12].

#### Gender profile

Consistent with previous findings of more coping engagement in women than men [34, 35, 38], the current study showed that women engaged in more coping strategies than men. Specifically, behavioural strategies were predominantly adopted by older women relative to any other age by gender groups. The results are largely consistent with earlier findings that women use strategies such as simply relaxing (e.g., Yoga) and staying socially connected to cope with the pandemic-associated stress [12, 38]. The higher engagement of coping in women might be driven by their overwhelmingly heightened negative emotions towards the pandemic [2, 4, 25], which urged them to actively cope [34, 35]. It may also reflect their higher emotional resilience than men when facing adversity [34, 65, 66]. Overall, women reported a higher coping endorsement than men, suggesting that women were more adaptive in coping with the pandemic-related stress despite their heightened negative emotional experiences. The results support the SAVI model [15] that assumes a higher resilience/strength among more vulnerable individuals.

In terms of age by gender intersectionality, the current study showed that the age-associated enhanced adaptive coping was more pronounced in women than men. Specifically, women experienced heightened negative emotion [e.g., [25]] and older adults showed stronger physical vulnerability to virus infection and social adversity during the lockdown. The current study also identified older women as most vulnerable to negative emotional experiences during the pandemic. These combined vulnerabilities might paradoxically make older women more adaptive in coping, possibly also driven by their strong motivation to maintain emotional wellbeing and their prominent social roles to maintain family and community cohesiveness [15, 20, 55]. The results also somewhat support the *Double Jeopardy Hypothesis* [57]. Specifically, combining two risk factors (young + man) led to most detrimental results (i.e., least adaptive in this case), whereas the opposite combination (old + woman) was associated with optimal adaptation.

### Limitations and conclusions

This study has several limitations. First, participants were recruited through a purposive sampling procedure via WeChat, the internet, and local Chinese communities. This approach may only reach those who are socially active, cognitively engaged, and psychologically motivated. Thus, the results may not be generalized to the broader Chinese Canadian population. Second, the interview was conducted through Zoom or phone by several research assistants. The results are mainly based on self-reported responses to relatively structured interview questions, which are likely subject to social desirability and interpretation bias that might differ across age and/or gender groups. However, we should point out that we took extensive measures to ensure rigour and consistency in the coding process by holding regular project meetings to resolve discrepancies. Methodological integrity was consistently monitored given the data collection and coding were primarily completed by well-trained research assistants who were blind to the questions and hypotheses of this study. Finally, we acknowledge that quantitative data analysis, particularly those 3-factor ANOVA models, may have limited statistical power due to the small sample size. Therefore, the results should be interpreted with caution and warrant further verification in future studies with a larger sample size. Nevertheless, it should be noted that the intention of the quantitative analysis was to provide preliminary descriptive statistics as supplementary to the primary qualitative results. Most importantly, the quantitative analysis focused on different aspects (i.e., frequency of emotional experiences, emotional words, and coping strategies) provided convergent patterns in consistency with the qualitative analysis. Therefore, we believe these analyses, despite limitations in statistical power, would effectively illustrate the patterns and trends observed within the respondents.

It should be highlighted that the results of the current study showed that the pandemic hits hard on women (given their overwhelming negative experiences) and young adults (given their lack of adaptive coping). However, both women and older adults are active and adaptive in coping, but probably for different reasons. Women were adaptive probably because of their practical need to regulate stress and negative emotions elicited by the pandemic. In contrast, older adults might effortfully reallocate their resources to strive for a positive mood driven by their goal to maintain emotional wellbeing. However, the results did not consider other potential covariates such as socioeconomic status, migration status, acculturation, education, financial and employment status. The conclusions might not be generalized beyond the socioeconomic characteristics of the specific samples in this study.

#### Implications and future directions

The current study represents a unique approach to systematically explore age and/or gender differences in emotional experiences and coping among Chinese migrants in Canada using a large-scale semi-structured qualitative interview. It makes an insightful and meaningful contribution to literature. The results shed important light on our understanding of psychological impacts of the pandemic and related coping of a minoritized population in light of adversity.

The findings of this study have significant practical implications for public health interventions, community programs, and policy aimed at enhancing the wellbeing of Chinese migrants in Canada during a public health crisis. The differentiated emotional responses and coping strategies across age and gender groups indicate a need for more tailored supports and services. Specifically, the observation that women, particularly older women, experienced higher levels of negative emotions suggests that mental health interventions should take an age-appropriate and gender-sensitive approach. Additionally, younger adults demonstrated a lack of adaptive coping strategies, making them particularly vulnerable and in need of targeted interventions to support their emotional resilience. Taken together, the results shed light on our understanding of the psychological impacts of COVID-19 and inform the practice of culturally and contextually sensitive prevention or intervention to best support minority communities in Canada.

Future research is needed with a larger sample size to strengthen the quantitative statistical power to ensure internal reliability of the results. Additionally, future research could also take a further step to examine and identify critical factors that contribute to the emotional experiences and adaptive coping of vulnerable populations in adversity. Some recently emerged qualitative research methods, such as *Online Photovoice* (OPV), *Online Interpretative Phenomenological Analysis* (OIPA) and *Community-Based Participatory Research* (CBPR) approaches (e.g., [67–70], have an advantage to capture and describe the thoughts, feelings, and behaviors of people from their own unique perspectives and lived experiences with minimal manipulations. Future research could consider adopting such methods to address the same or similar topics with a minoritized population.

Taken together, a few important preliminary conclusions could be drawn from the results: 1) the pandemic emotional experience is predominantly negative and behavioural coping is most prevalent during the pandemic; 2) women reported more negative emotional experiences than men; 3) older adults, women particularly, engaged in more adaptive coping than other groups. Importantly, the results preliminarily identified women to be most vulnerable to the detrimental emotional impacts and younger age to be associated with less adaptation in coping among Chinese migrants in Canada.

## Supplementary Information

Supplementary Material 1.

## Data Availability

The interview materials and NVivo coding files could be retrieved from 10.17605/OSF.IO/YTQE5 [71].

## Abbreviations

ANOVA: Analysis of Variance
CBPR: Community-Based Participatory Research
CBT: Cognitive Behavioral Therapy
CIHR: Canadian Institute of Health Research
COVID-19: Coronavirus Disease 2019
ID: Identification
NFRF: New Frontiers Research Fund
OIPA: Online Interpretative Phenomenological Analysis
OPV: Online Photovoice
QR code: Quick Response code
RA: Research Assistant
REB: Research Ethics Board
SAVI: Strength and Vulnerability Integration
SST: Socioemotional Selectivity Theory
U.K.: United Kingdom
U.S.: United States

## References

[1] Saladino V, Algeri D, Auriemma V. The psychological and social impact of Covid-19: new perspectives of well-being. Front Psychol. 2020;11:577–684. 33132986 10.3389/fpsyg.2020.577684 PMC7561673

[2] Browning M, Larson L, Sharaievska I, Rigolon A, McAnirlin O, Mullenbach L, et al. Psychological impacts from COVID-19 among university students: risk factors across seven states in the United States. PLoS ONE. 2021;16(1):e0245327. 33411812 10.1371/journal.pone.0245327 PMC7790395

[3] Na L, Yang L. Psychological and behavioral responses during the COVID-19 pandemic among individuals with mobility and/or self-care disabilities. Disabil Health J. 2021;15(1):101216–101216. 34649808 10.1016/j.dhjo.2021.101216 PMC8453786

[4] Persaud N, Woods H, Workentin A, Adekoya I, Dunn J, Hwang S, et al. Recommendations for equitable COVID-19 pandemic recovery in Canada. Can Med Assoc J CMAJ. 2021;193(49):E1878–88. 37578741 10.1503/cmaj.210904 PMC8677581

[5] Yang L, Yu L, Kandasamy K, Wang Y, Shi F, Zhang W, et al. Non-pathological psychological distress among mainland Chinese in Canada and its sociodemographic risk factors amidst the pandemic. HealthCare. 2022;10(11):2326. 36421650 10.3390/healthcare10112326 PMC9690647

[6] Yu L, Lecompte M, Zhang W, Wang PP, Yang L. Sociodemographic and COVID-related predictors for mental health condition of mainland Chinese in Canada amidst the pandemic. Int J Environ Res Public Health. 2022;19(1):171. 10.3390/ijerph19010171 PMC8750305 35010431

[7] Pfefferbaum B, North C. Mental health and the Covid-19 pandemic. N Engl J Med. 2020;383(6):510–2. 32283003 10.1056/NEJMp2008017

[8] Vigo D, Patten S, Pajer K, Krausz M, Taylor S, Rush B, et al. Mental health of communities during the COVID-19 pandemic. Can J Psychiatry. 2020;65(10):681–7. 32391720 10.1177/0706743720926676 PMC7502878

[9] Shepell M. Spotlight on the mental health impact of the COVID-19 pandemic. 2020. Available from: https://www.morneaushepell.com/sites/default/files/assets/permafiles/92440/mental-health-index-report-apr-2020.pdf.

[10] Settersten RA, Bernardi L, Härkönen J, Antonucci TC, Dykstra PA, Heckhausen J, et al. Understanding the effects of Covid-19 through a life course lens. Adv Life Course Res. 2020;45:100360. 36698274 10.1016/j.alcr.2020.100360 PMC7375263

[11] Ahmed A, Aqeel M, Aslam N. COVID-19 health crisis and prevalence of anxiety among individuals of various age groups: a qualitative study. J Ment Health Train Educ Pract. 2021;16(1):58–66.

[12] Finlay JM, Kler JS, O’Shea BQ, Eastman MR, Vinson YR, Kobayashi LC. Coping during the COVID-19 pandemic: a qualitative study of older adults across the United States. Front Public Health. 2021;9:643807. 33898379 10.3389/fpubh.2021.643807 PMC8058195

[13] García-Portilla P, de la Fuente TL, Bobes-Bascarán T, Jiménez Treviño L, Zurrón Madera P, Suárez Álvarez M, et al. Are older adults also at higher psychological risk from COVID-19? Aging Ment Health. 2021;25(7):1297–304. 32870024 10.1080/13607863.2020.1805723

[14] Yang L, Yu L, Zhang, W, Wei X, Shi F, Wang, PP. The perceived psychological distress towards COVID-19 and its demographic predictors. Ann Epidemiol. 2020;52:107–107.

[15] Carney AK, Graf AS, Hudson G, Willson E, Meeks S. Age moderates perceived COVID-19 disruption on well-being. Gerontologist. 2021;61(1):30–5. 32808660 10.1093/geront/gnaa106 PMC7454676

[16] Carstensen L, Shavit YZ, Barnes JT. Age advantages in emotional experience persist even under threat from the COVID-19 pandemic. Psychol Sci. 2020;31(11):1374–85. 33104409 10.1177/0956797620967261 PMC13171095

[17] Fuller HR, Huseth-Zosel A. Lessons in resilience: initial coping among older adults during the COVID-19 pandemic. Gerontologist. 2021;61(1):114–25. 33136144 10.1093/geront/gnaa170 PMC7665461

[18] Jiménez MP, Rieker JA, Reales RM, Ballesteros S. COVID-19 peritraumatic distress as a function of age and gender in a Spanish sample. Int J Environ Res Public Health. 2021;18(10):52–3. 10.3390/ijerph18105253 PMC8155941 34069224

[19] Zach S, Zeev A, Ophir M, Eilat-Adar S. Physical activity, resilience, emotions, moods, and weight control of older adults during the COVID-19 global crisis. Eur Rev Aging Phys Act. 2021;18(1):1–8. 33648448 10.1186/s11556-021-00258-w PMC7917372

[20] Na L, Yang L, Mezo PG, Liu R. Age disparities in mental health during the COVID19 pandemic: the roles of resilience and coping. Soc Sci Med. 2022;305:115031–115031. 35649300 10.1016/j.socscimed.2022.115031 PMC9100296

[21] Beam CR, Kim AJ. Psychological sequelae of social isolation and loneliness might be a larger problem in young adults than older adults. Psychol Trauma. 2020;12(S1):s58-60. 32525372 10.1037/tra0000774

[22] Whitehead BR, Torossain E. Older adults’ experience of the COVID-19 pandemic: a mixed-methods analysis of stresses and joys. Gerontologist. 2021;61(1):36–47. 32886764 10.1093/geront/gnaa126 PMC7499618

[23] Yang L, Lee ADY, Dong L. Psychological wellbeing and life satisfaction among Chinese older immigrants in Canada across the early and late stages of the COVID-19 pandemic. Healthc Basel. 2024;12(18):1899. 10.3390/healthcare12181899 PMC11431511 39337240

[24] Rahman MA, Hoque N, Alif SM, Salehin M, Islam SMS, Banik B, et al. Factors associated with psychological distress, fear and coping strategies during the COVID-19 pandemic in Australia. Glob Health. 2020;16(1):95–95. 10.1186/s12992-020-00624-w PMC7542573 33032629

[25] Choi I, Kim JH, Kim N, Choi E, Choi J, Suk HW, et al. How COVID-19 affected mental well-being: An 11- week trajectories of daily well-being of Koreans amidst COVID-19 by age, gender and region. PLoS ONE. 2021;16(4):e0250252–e0250252. 33891642 10.1371/journal.pone.0250252 PMC8064534

[26] Crenshaw K. Demarginalizing the intersection of race and sex: a black feminist critique of antidiscrimination doctrine, feminist theory and antiracist politics. Univ Chic Leg Forum. 1989;(1). Available from: http://chicagounbound.uchicago.edu/uclf/vol1989/iss1/8.

[27] Sanchez-Ruiz MJ, Tadros N, Khalaf T, Ego V, Eisenbeck N, Carreno DF, et al. Trait emotional intelligence and wellbeing during the pandemic: the mediating role of meaning-centered coping. Front Psychol. 2021;12:648401–648401. 34054650 10.3389/fpsyg.2021.648401 PMC8155707

[28] Gustems-Carnicer J, Calderon C. Coping strategies and psychological well-being among teacher education students: coping and well-being in students. Eur J Psychol Educ. 2013;28(4):1127–40.

[29] Emery RL, Johnson ST, Simone M, Loth KA, Berge JM, Neumark-Sztainer D. Understanding the impact of the COVID-19 pandemic on stress, mood, and substance use among young adults in the greater Minneapolis-St. Paul area: findings from project EAT. Soc Sci Med. 2021;276:113826–113826. 10.1016/j.socscimed.2021.113826 PMC8058317 33743209

[30] Vannini P, Gagliardi GP, Kuppe M, Dossett ML, Donovan NJ, Gatchel JR, et al. Stress, resilience, and coping strategies in a sample of community-dwelling older adults during COVID-19. J Psychiatr Res. 2021;138:176–85. 33862301 10.1016/j.jpsychires.2021.03.050 PMC8369528

[31] Lazarus RS. Coping theory and research: past, present, and future. Psychosom Med. 1993;55(3):234–47. 8346332 10.1097/00006842-199305000-00002

[32] Szabo C, Pukanszky J, Kemeny L. Psychological effects of the COVID-19 pandemic on Hungarian adults. Int J Environ Res Public Health. 2020;17(25):9565. 33371250 10.3390/ijerph17249565 PMC7766563

[33] Garcini LM, Rosenfeld J, Kneese G, Bondurant RG, Kanzler KE. Dealing with distress from the COVID-19 pandemic: mental health stressors and coping strategies in vulnerable latinx communities. Health Soc Care Community. 2022;30(1):284–94. 33894080 10.1111/hsc.13402 PMC8251305

[34] Fluharty M, Fancourt D. How have people been coping during the COVID-19 pandemic? Patterns and predictors of coping strategies amongst 26,016 UK adults. BMC Psychol. 2021;9(1):107–107. 34266498 10.1186/s40359-021-00603-9 PMC8280648

[35] Hennekam S, Shymko Y. Coping with the COVID-19 crisis: force majeure and gender performativity. Gend Work Organ. 2020;27(5):788–803. 32837010 10.1111/gwao.12479 PMC7280698

[36] Almomamni EY, Qablan AM, Almomany AM, Atrooz FY. The coping strategies followed by university students to mitigate the COVID-19 quarantine psychological impact. Curr Psychol. 2021;40(11):5772–81. 33994758 10.1007/s12144-021-01833-1 PMC8106545

[37] Prowse R, Sherratt F, Abizaid A, Gabrys RL, Hellemans KGC, Patterson ZR, et al. Coping with the COVID-19 pandemic: examining gender differences in stress and mental health among university students. Front Psychol. 2021;12:650759. 10.3389/fpsyt.2021.650759 PMC8058407 33897499

[38] Baloch GM, Kamaludin K, Chinna K, Sundarasen S, Nurunnabi M, Khoshaim HB, et al. Coping with COVID-19: the strategies adapted by Pakistani students to overcome implications. Int J Environ Res Public Health. 2021;18(4):1799. 33673237 10.3390/ijerph18041799 PMC7918213

[39] Tai DBG, Shah A, Sia IG, Wieland ML. The disproportionate impact of COVID-19 on racial and ethnic minorities in the United States. Clin Infect Dis. 2021;72(4):703–6. 32562416 10.1093/cid/ciaa815 PMC7337626

[40] Lin Z, Liu H, Kelley J. National study of racial-ethnic differences in COVID-19 concerns among older americans: evidence from the health and retirement study. J Gerontol. 2022;77(7):e134–41. 10.1093/geronb/gbab171 PMC9255937 34549286

[41] Nguyen LH, Anyane-Yeboa A, Klaser K, Merino J, Drew DA, Ma W, et al. The mental health burden of racial and ethnic minorities during the COVID-19 pandemic. PLoS ONE. 2022;17(8):e0271661. 35947543 10.1371/journal.pone.0271661 PMC9365178

[42] Statistics Canada. The Canadian census: a rich portrait of the country’s religious and ethnocultural diversity. 2022. Available from: https://www150.statcan.gc.ca/n1/en/daily-quotidien/221026/dq221026b-eng.pdf?st=Qoffokmg.

[43] Lee ADY, Wang P, Zhang W, Yang L. COVID-19 peritraumatic distress and loneliness in Chinese residents in North America: the role of contraction worry. Int J Environ Res Public Health. 2022;19(13):7639. 35805295 10.3390/ijerph19137639 PMC9265493

[44] Yu L, Yang L. Tracking the trajectory and predictors of peritraumatic distress among Chinese migrants in Canada across the three years of the COVID-19 pandemic. COVID. 2024;4(10):1642–54.

[45] Yang L, Kandasamy K, Na L, Zhang W, Wang P. Perceived and experienced anti-Chinese discrimination and its associated psychological impacts among Chinese Canadians during the wave 2 of the COVID-19 pandemic. Psychol Health Med. 2022;29(1):108–25. 36336783 10.1080/13548506.2022.2142947

[46] Chen JA, Zhang E, Liu CH. Potential impact of COVID-19-related racial discrimination on the health of Asian Americans. Am J Public Health. 2020;110(11):1624–7. 32941063 10.2105/AJPH.2020.305858 PMC7542280

[47] Hahm HC, Ha Y, Scott JC, Wongchai V, Chen JA, Liu CH. Perceived COVID-19-related anti-Asian discrimination predicts post traumatic stress disorder symptoms among Asian and Asian American young adults. Psychiatry Res. 2021;303:114084. 34242971 10.1016/j.psychres.2021.114084 PMC8661065

[48] Bresnahan M, Zhu Y, Hipple S, Savoie L, Corrigan PW. The negative health effects of anti-Asian stigma in the U.S. during COVID-19. Stigma Health. 2022;8(1):115–23.

[49] Gao Z. Unsettled belongings: Chinese immigrants’ mental health vulnerability as a symptom of international politics in the COVID-19 pandemic. J Humanist Psychol. 2021;61(2):198–218.

[50] Kobayashi A, Preston V. Being CBC: the ambivalent identities and belonging of Canadian-born children of immigrants. Ann Assoc Am Geogr. 2014;104(2):234–42.

[51] Mojaverian T, Kim HS. Interpreting a helping hand: cultural variation in the effectiveness of solicited and unsolicited social support. Pers Soc Psychol Bull. 2013;39(1):88–9. 23131905 10.1177/0146167212465319

[52] Mao Y. Investigating Chinese migrants’ information-seeking patterns in Canada: media selection and language preference. Glob Media J Can Ed. 2015;8(2):113–31.

[53] Tieu Y, Konnert CA. Mental health help-seeking attitudes, utilization, and intentions among older Chinese immigrants in Canada. Aging Ment Health. 2014;18(2):140–7. 23837711 10.1080/13607863.2013.814104

[54] Chen AW, Kazanjian A, Wong H. Why do Chinese Canadians not consult mental health services: health status, language or culture? Transcult Psychiatry. 2009;46(4):623–41. 20028680 10.1177/1363461509351374

[55] Carstensen LL. Socioemotional selectivity theory: the role of perceived endings in human motivation. Gerontologist. 2021;61(8):1188–96. 34718558 10.1093/geront/gnab116 PMC8599276

[56] Reed AE, Chan L, Mikels JA, Mayr U. Meta-analysis of the age-related positivity effect: age differences in preferences for positive over negative information. Psychol Aging. 2014;29(1):1–15. 24660792 10.1037/a0035194

[57] Chappell NL, Havens B. Old and female: testing the double jeopardy hypothesis. Sociol Q. 1980;21(2):157–71.

[58] Berg BL, Lune H. Qualitative research methods for the social sciences. Boston: Pearson; 2012.

[59] Wang SC, Santos BMC. “Go Back to China With Your (Expletive) Virus”: a revelatory case study of anti-asian racism during COVID-19. Asian Am J Psychol. 2022;13(3):220–33.

[60] Braun V, Clarke V. Is thematic analysis used well in health psychology? A critical review of published research, with recommendations for quality practice and reporting. Health Psychol Rev. 2023;17:1–24. 36656762 10.1080/17437199.2022.2161594

[61] Clarke V, Braun V. Thematic analysis. J Posit Psychol. 2017;12(3):297–8.

[62] Schreier M. Qualitative content analysis in practice. 2012. 171–183 p.

[63] Al-Tammemi AB, Amal A. Is it just about physical health? An online cross-sectional study exploring the psychological distress among university students in Jordan in the midst of COVID-19 pandemic. Front Psychol. 2020;11:562213–562213. 33240151 10.3389/fpsyg.2020.562213 PMC7677563

[64] Gray J. Men Are from Mars, Women Are from Venus : a Practical Guide for Improving Communication and Getting What You Want in Your Relationships. New York, NY: HarperCollins, 1992.

[65] Ferreira RJ, Adolph V, Hall M, Buttell F. Predictors of individual resilience: gender differences among African Americans. J Evid-Based Soc Work. 2019;16(4):347–62.

[66] Mwangi NC. Gender differences in academic resilience and academic achievement among secondary school students in Kiambu county. Kenya Psychol Behav Sci Int J. 2017;5(5):1–7.

[67] Dari T, Chan CD, Del Re J. Integrating culturally responsive group work in schools to foster the development of career aspirations among marginalized youth. J Spec Group Work. 2021;46(1):75–89.

[68] Dari T, Fox C, Laux JM, Speedlin GS. The development and validation of the Community-Based Participatory Research Knowledge Self-Assessment Scale (CBPR-KSAS): a Rasch analysis. Meas Eval Couns Dev. 2023;56(1):64–79.

[69] Tanhan A, Strack RW. Online photovoice to explore and advocate for Muslim biopsychosocial spiritual wellbeing and issues: Ecological systems theory and ally development. Curr Psychol. 2020;39(6):2010–25.

[70] Tanhan A. Utilizing Online Photovoice (OPV) methodology to address biopsychosocial spiritual economic issues and wellbeing during COVID-19: adapting OPV to Turkish. Turk Stud. 2020;15(4):1029–86.

[71] Lab. Psychological impacts of COVID-19: interview (2020). 2021.

