# Supplementary material for: An age and gender stratified interview on emotional experiences and coping of Chinese migrants in Canada amidst the pandemic
Source: BMC Psychol. 2025 Jul 4;13:735. doi: 10.1186/s40359-025-02993-6 (PMC12228377; doi:10.1186/s40359-025-02993-6)
Supplement: Supplementary file 1 — Supplementary Material 1. [file 40359_2025_2993_MOESM1_ESM.docx]

**Appendix 1.** Interview Script

**Greeting Instruction:**

XXX , hello, thank you for signing up for this interview on the impact of the COVID-19 by a research team from XXX institutes. [For Zoom Interview] To ensure a smooth and interactive interview, please feel free to turn on your video camera. For research purpose, the interview will be recorded. Do you have any questions before we start?  [address questions if any]

You probably have read the consent form. Do you need me to quickly go over the main points?

[if “YES”] great! the purpose of this interview is to examine the psychological impact of the COVID-19 on Chinese immigrants in Canada. The participation is entirely voluntary. All the information collected will be kept confidential. You can withdraw at any time point or refuse to answer any questions you do not feel comfortable to answer.

[if “NO”] start the interview.

If you do not have any other questions, we will start the interview now. Now I start recording.

**[Start recording]**

Based on the information in the consent form, would you please let me know whether you consent to participate? If yes, please response “Yes, I consent to participate”. We need your audio recorded consent to indicate that we have your consent to participate in this interview. Thank you!

**Interview Instruction:** Thank you for participating in this interview. I will now ask you a few questions which may not have standard right or wrong answers. Please try to respond in details based on your personal experiences. All the information collected from you will be kept strictly confidential. You can withdraw at any time point or refuse to answer any questions you do not feel comfortable to answer. Just let me know. Thank you!

**Personal information**

1. Basic background information [Google form or phone registration questions, only check if any information provided during the registration is not clear. Otherwise, skip this item]

**Age (); Gender :**Female ; Male ; Other

**Marital status :**single ; married/partnered ; divorced / separated ; widowed

**Highest education:**elementary school and below ; junior high school ; high school; college ; university; postgraduate and above

**Employment status :**Retired ; full-time students ; full-time employed ( medical workers) ; full-time employed ( other areas) ; contract / part-time job; self-employed ; farmers ; unemployed (including lay-off or housewives , etc. )

**Religion :**No ; Christianity ; catholic ; Islam ; Buddhism ; other ()

**Your immigration status in Canada :**citizen ; permanent resident ; international student ; visiting/touring ; business ; other ()

**How long have you been in Canada:**() year () month

1. Financial situation: Are you satisfied with your current financial situation?
2. What do you think is the difference between Canadian and Chinese culture? Which culture do you identify with?
3. Which region do you live in Canada?

**COVID-19 Experiences:**

1. Please describe how your daily life (e.g., work/study/family activities, behavioural routines and socialization) is affected by the COVID-19 pandemic?
2. Please describe how your future life/career/education goals might change as a result of the COVID-19 pandemic?
3. Please describe how the life of your family, friends, and relatives (your close ones) is affected by the COVID-19 pandemic? (For example, work/learning/family activities, behaviour habits and social aspects, specially time arrangement every day, is there any difference from before)
4. Please describe how Canada in general specially is impacted by the COVID-19 pandemic?
5. Please describe how Chinese community specifically is affected by the COVID-19 pandemic?
6. Do you feel that you are well-supported during the COVID-19 outbreak? Please describe the support you typically receive from others (e.g., family, friends/relatives, the community, the country) during the COVID-19 outbreak?
7. How do you feel about the policies and strategies that Canada government and/or Public Health has implemented to deal with the COVID-19 spread? Is there anything else you think could have been done to best control this pandemic?

**Psychological impact and coping**

1. Please list your top concerns/worries about the COVID-19 pandemic?
2. Please describe how you usually receive the information (e.g., sources, reliability, and amount) about the COVID-19?
3. Please describe your overall feelings or emotions towards the COVID-19 outbreak?
4. What strategies have you used to regulate your fear and concern about the COVID-19?
5. How have you helped others (e.g., family, friends, or others) cope with their fears and concerns about the COVID-19?
6. Please describe your practice to take care of the mental and physical health of yourself and close others during the COVID-19 pandemic?
7. What mental health coping strategies or resources do you wish to have during this time?
8. Have you experienced stigma and discrimination against Chinese or Chinese community in Canada as a result of the COVID-19? Please explain?
